# Drone‐Assisted Organ Transport: A Scoping Review of Clinical, Regulatory, and System Readiness

**DOI:** 10.1111/ctr.70398

**Published:** 2025-12-11

**Authors:** Robson G. Gilmour, Mekhola Hoff

**Affiliations:** ^1^ University of Edinburgh Edinburgh UK; ^2^ Royal Infirmary of Edinburgh Edinburgh Transplant Centre Edinburgh UK

**Keywords:** donors and donation, economics, organ perfusion and preservation, organ procurement, transplant coordinator

## Abstract

Organ transport remains a critical determinant of transplant success, with delays during transfer prolonging cold ischemia time (CIT) and increasing the risk of delayed graft function or organ discard. Uncrewed aerial vehicles (UAVs) have emerged as a potential means to enhance the speed, predictability, and resilience of transplant logistics. This scoping review synthesized global evidence from 2010 to 2025 across biomedical, engineering, and regulatory domains, mapping research within six key themes: time sensitivity and clinical evidence, integration with preservation and perfusion technologies, technological and infrastructural readiness, regulatory and ethical frameworks, economic and environmental feasibility, and public and professional preparedness. Case studies from North America, Europe, and Asia demonstrate that UAVs can safely deliver donor organs while maintaining temperature and structural stability, with successful clinical transplantation reported. Their current value lies primarily in short‐range transfers where direct routing and rapid handover are feasible; however, advances in heavy‐lift aircraft, autonomous traffic management, and connected perfusion systems may soon enable longer, more complex missions. Major barriers include restrictions on beyond‐visual‐line‐of‐sight (BVLOS) operation, payload and endurance limitations, uncertain liability frameworks, and incomplete hospital‐side infrastructure. Future priorities include comparative clinical and economic trials, harmonized international standards, and certified medical air corridors integrated with national allocation systems. With appropriate regulation, interoperability, and clinical validation, drones could become a safe and sustainable extension of existing transplant networks—improving efficiency, equity, and ultimately, patient survival.

**Abbreviations**
AES‐256: advanced encryption standard (256‐bit)
AI: artificial intelligence
API: application programming interface
BVLOS: beyond visual line of sight
CAA: (UK) Civil Aviation Authority
CAELUS: Care & Equity—Healthcare Logistics UAS (Scotland program)
CAP: civil aviation publication
CIT: cold ischemia time
CO_2_
: carbon dioxide
DGF: delayed graft function
EMR: electronic medical record
FAA: (US) Federal Aviation Administration
FDA: (US) Food and Drug Administration
GDPR: General Data Protection Regulation (EU)
GPS: global positioning system
HIPAA: Health Insurance Portability and Accountability Act (US)
HLA: human leukocyte antigen
HMP/NMP: hypothermic/normothermic machine perfusion
HOMAL: human organ monitoring and quality apparatus for long‐distance travel
HRSA: (US) Health Resources and Services Administration
HTA: Human Tissue Authority (UK)
IMU: inertial measurement unit
IoT: internet of things
NHS: National Health Service (UK)
NHSBT: NHS Blood and Transplant (UK)
NOMOH: “Not Over My Own Home” (public acceptance effect)
OCS: Organ Care System (TransMedics)
OPO: Organ Procurement Organization
PPP: public–private partnership
RFID: radio‐frequency identification
SAEAR: serious adverse event and reaction
SC‐VTOL: small‐category vertical take‐off and landing
SOP: standard operating procedure
TLS 1.3: Transport Layer Security (version 1.3)
UAS/UAV: uncrewed aircraft system/uncrewed aerial vehicle
UKRI: UK Research and Innovation
UNOS: United Network for Organ Sharing (US)
UTM: uncrewed traffic management
VLOS: visual line of sight
VTOL/eVTOL: vertical take‐off and landing/electric VTOL

## 1 Introduction

Organ transplantation remains among medicine's most powerful interventions, but it is defined by one inescapable constraint: time. Though over 172 000 solid organ transplants were reported globally in 2023, many more patients continue to wait [1]. In the United States alone, more than 100 000 patients remain on the waiting list, with mortality continuing to rise [2].

Once an organ is offered, its journey from donor to recipient becomes a tightly choreographed sequence—retrieval, preservation, transport, implantation—where transport often poses the greatest logistical hazard. As allocation policies evolve toward wider geographic sharing, organs now routinely travel greater distances [3, 4]. Transport logistics, therefore, exert a direct influence on graft viability and transplant outcomes; later sections quantify these effects.

Conventional transport modalities carry inherent vulnerabilities. A 2009 US survey reported that 21% of organ transports used fixed‐wing aircraft, 16% personal vehicles, 14% ambulances, and 5% helicopters [3]. Ground couriers face congestion and scheduling constraints, helicopters are weather‐sensitive and costly, and fixed‐wing flights add airport handling and coordination time. Safety is another concern: non‐commercial procurement flights account for most transplant‐related aviation incidents, with fatality risk reported as markedly higher than for commercial aviation (Scalea et al. 2019). These vulnerabilities disproportionately affect marginal organs and those with narrow ischemic tolerance.

Uncrewed aerial vehicles (UAVs) now present a credible adjunct to existing transport chains. Once confined to defense and industrial applications, UAV technology has matured into a versatile civil platform offering precision, reliability, and automation. Multi‐rotor drones enable vertical take‐off for short‐range urban transfers, fixed‐wing models provide efficient long‐distance flight, and hybrid VTOL systems combine both capabilities—each suited to different segments of the transplant pathway.

Their reliability in healthcare logistics is already well established: Rwanda and Ghana operate national drone networks for blood, vaccines, and diagnostics [5, 6]. In higher‐income settings, programs such as CAELUS in Scotland and Apian in England are piloting hospital‐to‐hospital corridors within regulated frameworks [7, 8]. Table 1 summarizes major medical‐drone programs currently in operation worldwide, illustrating how UAV logistics are transitioning from pilot studies to integrated health‐system use [9].

**TABLE 1:** Key global medical drone delivery projects (2015–2024).

Year	Country	Company/Initiative	Payload type	Reported distance	Payload capacity	Max drone speed	Approx. flight duration	Notes/Highlights
2015	USA (Virginia)	Flirtey [10]	Emergency drugs	0.8 km	4.5 kg	Not specified	3 min	First FAA‐approved drone delivery in the US. Delivered medical supplies to a rural health clinic.
2016	Rwanda	Zipline [11]	Blood products	50km	Up to 1.8 kg	∼100 km/h	∼14 min	First national drone delivery service, significantly cut blood delivery time.
2017	Sweden	Karolinska Institute [12]	AEDs	∼10 km	Not specified	Not specified	∼5 min	Drones delivered AEDs faster than ambulances.
2017	Switzerland	Swiss Post + Matternet [13]	Blood, lab samples	20 km	2 kg	Not specified	17 min	Routine transport between hospitals and labs in Lugano.
2018	Vanuatu	UNICEF [14]	Vaccines	Up to 47 km	Not specified	Not specified	Not specified	Delivered vaccines to remote islands, improved access.
2019	Ghana	Zipline	Vaccines, blood, meds	Up to 80 km	Up to 1.8 kg	∼100 km/h	∼45 min	Expanded Zipline's success in Rwanda to broader operations.
2020	USA (North Carolina)	Zipline/Novant Health	PPE, medical supplies	Up to 80 km	Up to 1.8 kg	∼100 km/h	∼45 min	FAA‐approved long‐range COVID‐19 drone delivery.
2021	Botswana	Avy [15]	Medical supplies	Up to 60 km	Up to 1.5 kg	∼74 km/h	∼55 min	Reduced transport time by 65% vs. road.
2022	Nigeria	Zipline	Vaccines, medical supplies	Up to 80 km	Up to 1.8 kg	∼100 km/h	∼45 min	Expanding reliable medical delivery network.
2022	UK (Isle of Wight)	NHS/Apian [16]	Chemotherapy drugs	47 km	Not specified	Not specified	30 min	Saved hours by replacing car/ferry transport.
2023	UK (Scotland)	Project CAELUS	Medical supplies	∼50 km	Not specified	Not specified	35 min	Among the first UK trials to demonstrate BVLOS operations transitioning between controlled and uncontrolled airspace.
2024	UK (London)	NHS/Apian	Blood samples	∼2 km	Not specified	∼100 km/h	<2 min	Slashed delivery time from 30+ minutes to under 2.

*Note:* Compiled from sources including peer‐reviewed literature, government reports, NGO publications, and reputable media sources. Full references are listed at the end of the manuscript.

These initiatives collectively demonstrate growing regulatory legitimacy, technical maturity, and operational precedent on which transplant UAV deployment can build. However, widespread implementation remains limited by incomplete evidence, fragmented regulation, and uncertain system readiness.

The aim of this scoping review is to synthesize global evidence on drone‐assisted organ transport, evaluating its clinical feasibility, technological maturity, regulatory preparedness, and system integration, and to identify the key gaps that must be addressed for routine clinical adoption.

## 2 Methodology

The aim of this review was to map and critically synthesize current global evidence on drone‐assisted organ transport, evaluating its clinical feasibility, technological readiness, regulatory preparedness, and system‐level integration. To achieve this, the study was designed as a clinical scoping review, chosen for its suitability in emerging, interdisciplinary fields where the evidence base is heterogeneous and not yet amenable to quantitative meta‐analysis.

The review sought to identify key themes, implementation priorities, and evidence gaps across six core domains:

1. Time Sensitivity and Clinical Evidence
2. Integration with Organ Perfusion Systems
3. Technological and Operational Integration
4. Regulatory, Legal, and Ethical Readiness
5. Economic and Environmental Considerations
6. Public and Professional Readiness

The purpose of this approach was to provide a comprehensive overview of the current state of research, highlight barriers to clinical adoption, and define future directions for experimental, regulatory, and policy development.

### 2.1 Search Strategy

Searches were performed between November 2024 and September 2025, covering literature published from 2010 to 2025.

Peer‐reviewed sources were identified from PubMed, ScienceDirect, IEEE Xplore, Embase, Web of Science, Scopus, SSRN, MedRxiv, and BioRxiv.

Gray literature searches were conducted across government, regulatory, and industry sources, including:

- Health and transplant bodies: NHS England, NHS Blood and Transplant (NHSBT), Human Tissue Authority (HTA), UK Civil Aviation Authority (CAA), US Federal Aviation Administration (FAA), UNOS/OPTN
- Industry and trial programs: Apian, CAELUS (Scotland), AGS Airports, Zipline, MissionGO, Avy, Matador UAS Consortium, and ABzero (Italy).
- Academic and healthcare institutions: University of Maryland Medical Center, Toronto General Hospital, and Guy's & St Thomas’ NHS Foundation Trust.
- Professional and technical sources: aviation white papers, conference proceedings, Nesta's *Flying High* reports, and relevant media coverage (BBC, CNBC, DroneLife).

Search terms combined UAV descriptors (“drone,” “unmanned aerial vehicle,” “uncrewed aerial vehicle,” “UAV”) with clinical and operational keywords (“organ transport,” “transplant logistics,” “cold ischemia,” “machine perfusion,” “organ perfusion,” “BVLOS,” “medical drone,” “tracking,” “telemetry,” “supply chain,” “time delay,” “regulation,” “airspace,” “policy,” “legal framework,” “cybersecurity,” “environmental impact”). Boolean operators were adapted for each database to optimize sensitivity and specificity. Reference lists of key studies and program reports were also manually searched.

### 2.2 Selection and Data Charting

All records were screened in two stages—title/abstract followed by full‐text—by the first author (R.G.G.), with uncertain inclusions discussed with the senior author (M.H.). Studies meeting the inclusion criteria were logged in a structured spreadsheet and categorized according to the six thematic domains of the review. Key characteristics such as study type, payload, flight context, and principal findings were recorded to facilitate comparison across domains. Peer‐reviewed and gray‐literature sources were then collated and synthesized thematically, allowing patterns, gaps, and areas of convergence to be identified across clinical, technical, regulatory, and system perspectives.

### 2.3 Inclusion and Exclusion Criteria

#### 2.3.1 Inclusion

- English‐language publications (2010–2025).
- Studies or reports directly addressing UAV use in healthcare logistics relevant to organ transport or other time‐critical medical payloads (e.g., blood, biopsy, perfusion devices, pharmacologic samples).

#### 2.3.2 Exclusion

- Military or defense applications.
- Non‐translated foreign‐language texts.
- Logistics studies without UAV or transplant relevance.

#### 2.3.3 Data Synthesis and Reporting

Due to study heterogeneity, findings were synthesized thematically across the six domains listed above, using time and clinical outcomes as integrative axes. Key real‐world case studies—including the Baltimore kidney, Toronto lung, Minnesota pancreas, and Turin kidney flights—illustrate practical implementation.

**FIGURE 1:**
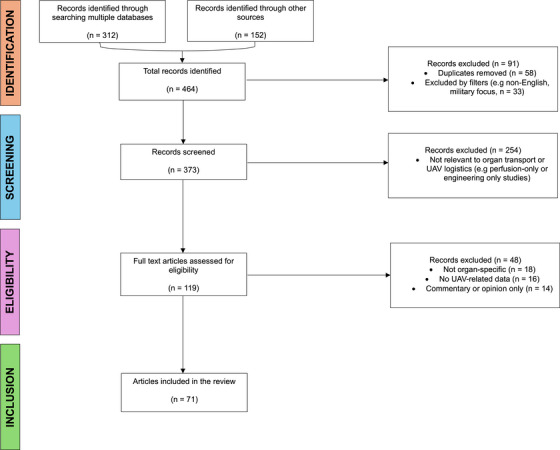
PRISMA‐ScR flow diagram. Flow diagram illustrating the literature search and inclusion process for the scoping review, conducted between November 2024 and September 2025. A total of 464 records were identified through database and gray‐literature searches, with 71 sources meeting inclusion criteria after screening and eligibility assessment. Reasons for exclusion are shown at each stage. The review followed PRISMA‐ScR guidelines.

The review followed PRISMA‐ScR (Preferred Reporting Items for Scoping Reviews) guidance. The search and inclusion process is shown in Figure 1 (PRISMA‐ScR Flow Diagram).

### 2.4 Limitations

No formal risk‐of‐bias assessment was performed. Restriction to English‐language and published or officially released reports may have excluded relevant non‐indexed data. The findings are descriptive and intended to guide future clinical trials, regulatory frameworks, and system‐design studies.

### 2.5 Time Sensitivity and Clinical Evidence for UAV‐Based Organ Transport

Time is the defining constraint in transplantation. Cold ischemia time (CIT)—the period during which an organ remains cooled and without blood supply—is one of the strongest predictors of graft outcome and the second most common reason for organ discard after intrinsic quality [2]. Observational data show a near‐linear relationship between prolonged CIT and poorer graft survival, with each additional hour of CIT significantly increasing the risk of graft failure and mortality in renal transplants [17].

Large‐scale registry analyses reinforce this vulnerability. In the United States, revised kidney‐sharing policies increased mean travel distance by around 73 miles, leading to a rise in DGF from 25% to 31% [3, 4]. Even modest 30–60‐min delays during transfer can push a graft from acceptable to marginal, particularly for hearts and lungs whose preservation limits are measured in minutes rather than hours. Typical tolerances range from 4 to 6 h for hearts, 6 to 8 h for lungs, less than 12 h for liver and pancreas, and up to 24 h for kidneys, beyond which graft dysfunction and discard rates increase sharply.

Transport delay is therefore one of the few modifiable determinants of transplant success. If a system can reliably reduce even a few minutes of transit time, it may meaningfully improve outcomes by converting marginal organs into viable grafts and preserving the narrow ischemic window that determines success.

Uncrewed aerial vehicles (UAVs) directly address this vulnerability by bypassing congestion, flight scheduling, and crew‐availability constraints to provide rapid, predictable, point‐to‐point transfer. Recent modeling studies quantify these potential gains. In an Austrian–German network analysis, Karpstein et al. [18] found that small UAVs could perform ≈37% of organ transports, saving 15–17 min on average compared with ground routes, while medium UAVs achieved savings exceeding 1 h in most scenarios. Larger SC‐VTOL aircraft demonstrated even greater potential, reducing total travel time by 60–90 min on longer routes. These data suggest UAV‐enabled mobility could meaningfully compress transfer times, particularly in cardiac and pulmonary transplantation, where every minute of CIT is critical.

Simulation and logistics‐modeling studies have indicated UAVs may achieve markedly faster response times—potentially several‐fold faster than ground transport in idealized conditions [19]. Based on typical preservation tolerances, clinically meaningful time savings would likely exceed 60 min for hearts and lungs, 30–60 min for livers, and 60–120 min for kidneys, though the precise threshold will depend on donor quality, graft perfusion status, and overall cold‐ischemia management—margins that current UAV systems are already capable of achieving under operational conditions.

Together, these findings position UAVs as a targeted, evidence‐driven solution to one of transplantation's few remediable constraints: time. The following section summarizes emerging clinical evidence from real‐world flights that have validated these principles under operational conditions.

### 2.6 Clinical Feasibility and Emerging Evidence

Over the past 5 years, demonstrations have confirmed that drones can safely and reliably transport organs under real‐world conditions, maintaining environmental stability and structural integrity throughout flight. Table 2 summarizes reported drone‐assisted organ delivery missions to date, including flight distance, payload type, and clinical outcomes.

**TABLE 2:** Selected UAV organ‐delivery demonstrations (2019–2024).

Year	Location	Organ(s)	Study type	Distance (km)	Flight time (min)	Outcome
2019	Baltimore, USA	Kidney	Clinical transplant	4.3	∼10	Delivered; transplanted; stable telemetry
2021	Toronto, CAN	Lung	Clinical transplant	1.5	6	Delivered; transplanted; uneventful recovery
2021	Minneapolis, USA	Pancreas	Research payload	16	∼20 loop	Biopsy unchanged pre/post
2020	Las Vegas, USA	Kidney	Research payload	16.6	∼20	Viability confirmed
2024	Turin, ITA	Kidney (sim)	Simulated payload	0.5	∼2	Autonomy + smart capsule validated

*Note:* Documented cases of organ or organ‐equivalent transport via uncrewed aerial vehicle (UAV), summarizing payload type, flight distance, environmental monitoring, and clinical outcome where applicable. Sources include peer‐reviewed publications and verified institutional reports (2019–2025).

In 2019, a custom UAV transported a human kidney 2.8 miles (≈4.5 km) across Baltimore to the University of Maryland Medical Center after more than 40 preparatory flights. Continuous telemetry via the Human Organ Monitoring and Quality Apparatus for Long‐Distance Travel (HOMAL) system verified stable temperature, pressure, and vibration levels during the 9.5‐min mission. The kidney was successfully transplanted—the world's first clinical drone organ delivery [20].

In 2021, MissionGO completed the first drone transport of a human pancreas in Minnesota, flying a 10‐mile (16 km) loop over the Mississippi River. Real‐time tracking confirmed environmental stability, and pre‐ and post‐flight biopsies showed no histological or biochemical change, demonstrating that even metabolically sensitive tissue could tolerate UAV carriage [4].

In 2022, Toronto surgeons achieved the first successful lung delivery by drone, completing a 1.5 km flight in approximately 5 min before transplanting the organ into a recipient with idiopathic pulmonary fibrosis. The lung arrived in excellent condition, and recovery was uneventful [21].

International efforts have since expanded this model. In Japan, drones transported rat livers 12 km without deterioration in enzyme profiles or histological integrity, confirming biochemical stability even in small‐scale models [22]. In 2024, an Italian consortium in Turin completed Europe's first autonomous urban organ‐transfer test, flying a kidney surrogate capsule 500 m between hospitals using a smart biocontainer with live temperature and humidity control [23]. In India, a collaboration led by MGM Healthcare demonstrated prototype drones capable of transporting hearts and kidneys [24].

Collectively, these demonstrations confirm that properly packaged and continuously monitored organs can withstand drone transport without compromising viability. UAV delivery also simplifies logistics by eliminating multiple handovers and enabling direct hospital‐to‐hospital transfer. Surgical teams can prepare in advance, often on the same rooftop or within a designated hospital pad, creating smoother integration with operative workflow.

### 2.7 From Feasibility to Measurable Value

While early results confirm the technical and biological feasibility of UAV organ delivery, the next challenge is demonstrating measurable value—quantifiable improvements in clinical efficiency, reliability, and cost compared with existing systems. To date, all published flights have involved short distances (<5 km) and single payloads under tightly controlled conditions. Current UAVs remain constrained by battery endurance, payload capacity, and regulatory limits on visual‐line‐of‐sight (VLOS) operation.

Accordingly, near‐term deployment is best suited to short‐hop transfers between donor hospitals, airports, and transplant centers—congestion‐prone segments of the transport chain where small time losses are most common. Scaling beyond this will require larger certified aircraft with redundant safety systems and permissions for beyond‐visual‐line‐of‐sight (BVLOS) operation.

A 2025 *Wall Street Journal* report described a planned US‐based clinical trial, expected around 2026 pending federal funding, that will directly compare drone and ground transport under real‐world conditions [25]. Such comparative studies will be pivotal in validating UAVs as a routine modality within regulated transplant pathways.

In the interim, lighter medical payloads—such as crossmatch specimens, HLA samples, and post‐transplant biopsies—offer an attractive proving ground [26]. These applications involve minimal biological risk and fewer regulatory barriers, yet still test the same core requirements of telemetry accuracy, environmental stability, and traceable custody. Demonstrating consistent reliability in these lower‐risk domains could accelerate regulatory acceptance and build professional confidence ahead of widespread clinical use.

## 3 Integration of Organ Perfusion Systems and UAV‐Based Logistics

Machine perfusion has transformed organ preservation, redefining how grafts are maintained and transported. Cold static storage remains the global standard because of its simplicity, portability, and low cost, yet it provides no metabolic support and imposes strict temporal limits on retrieval distance and scheduling. Portable perfusion devices now maintain active circulation and allow functional assessment before implantation, shifting transplantation from a race against time to a process that can be optimized and monitored.

Two principal techniques—hypothermic machine perfusion (HMP) and normothermic machine perfusion (NMP)—have shown consistent benefit. HMP circulates oxygenated perfusate at 4°C–10°C to suppress metabolism, while NMP maintains near‐physiological temperature and organ function, enabling real‐time viability testing and even reconditioning of marginal grafts. Systems such as the TransMedics Organ Care System (OCS) and XVIVO Liver Assist have demonstrated safety and efficacy across multiple organs [27]. In the OCS Liver PROTECT trial, NMP reduced early allograft dysfunction compared with static storage (17.3% vs. 30.5%) and increased utilization of extended‐criteria grafts [28]. Comparable outcomes were reported in the OCS Heart (PROCEED II) and INSPIRE Lung trials [29, 30]. For kidneys, meta‐analyses confirm that HMP reduces delayed graft function and improves early performance, while experimental studies have maintained liver viability beyond 20 h and, in select cases, for several days [31, 32].

Drones, or uncrewed aerial vehicles (UAVs), complement perfusion by targeting the same constraint—time—from the opposite direction. UAVs provide direct, predictable transfer that complements extended preservation. Together they form a synergistic framework for optimizing both preservation and delivery.

Current perfusion consoles typically weigh 20–100 kg and require manual handling, exceeding the payload capacity of most medical drones. Lightweight kidney systems such as the LifePort (∼20 kg) and XVIVO Kidney Assist Transport (∼25 kg) sit near the upper limit of current UAV capability. [33] However, advances in heavy‐lift hybrid‐electric drones—already proven in defense and industrial logistics—indicate that airborne carriage of organs during perfusion is technically feasible. Once appropriate safety and certification frameworks are established, autonomous transport of perfused grafts could substantially reduce the logistical pressures that have long defined organ transport.

Modern perfusion platforms also include telemetry functions that enable continuous monitoring of temperature, flow, and pressure during transport. When linked with UAV flight data, these systems allow teams to confirm environmental stability and organ status throughout transfer. Broader applications for integrated tracking and data exchange are discussed in the following section.

Ultimately, combining portable machine perfusion with UAV delivery offers a credible pathway toward safer and more reliable transplant logistics. It unites two complementary advances—active preservation and autonomous transport—capable of extending access to transplantation while maintaining rigorous standards of graft quality and clinical oversight.

## 4 Technological and Operational Integration of UAV Systems

Drone‐based organ transport remains in the proof‐of‐concept stage, with several demonstration flights validating technical feasibility under controlled conditions. While these missions have confirmed stability, safety, and environmental monitoring, sustained clinical use within regulated transplant logistics has not yet been achieved. The main challenge is therefore translational—how to move from experimental demonstrations to reliable, integrated deployment within time‐critical transplant pathways while maintaining the safety, reliability, and traceability expected in clinical care. Achieving this will require coordinated advances in technology, infrastructure, and operational frameworks capable of supporting real‐world, high‐frequency use.

### 4.1 In‐Flight Stability and Environmental Monitoring

Donor organs are highly sensitive to vibration, temperature, and pressure fluctuations during transport, making environmental stability a key determinant of post‐transplant function. The first systematic investigation came from the University of Maryland HOMAL study, which monitored temperature, barometric pressure, vibration, and GPS during 14 unmanned kidney transport missions [34]. Across flights up to 3 miles, mean organ temperatures remained stable at ≈2.5°C, barometric pressure changes correlated predictably with altitude, and vibration forces were <0.5 G—far below the >2 G recorded in fixed‐wing aircraft. Biopsies confirmed no histological injury, establishing that short‐range UAV transport can maintain both structural and physiological stability. Complementary work from Norway modeled high‐volume sample logistics and found that whole blood tolerates UAV vibration exposure, whereas plasma is more susceptible to separation at higher g‐forces [35, 36].

Subsequent engineering work refined this foundation. Bhayje et al. [37] quantified relationships between speed, payload, and stability, showing that quadcopters achieve optimal performance for loads ≤1.2 kg while maintaining precise hover control with minimal roll‐pitch deviation. This stability depends primarily on the drone's onboard inertial‐measurement units (IMUs), which auto‐correct micro‐movements, and on symmetrical motor design that limits vibration transmission to the payload.

Advances in thermal regulation have further enhanced UAV viability for biomedical transport. Sankaran et al. [38] developed a smart IoT container maintaining internal temperatures between 4°C and 12°C for 6–8 h, transmitting live telemetry to operators. Pamula and Ramachandran [39] demonstrated a hybrid system combining phase‐change materials with thermoelectric modules to sustain ±0.5°C precision while conserving battery life. Together, these studies confirm that precise temperature and vibration control are technically feasible in autonomous organ transport.

Integrating payload telemetry (temperature, humidity, and vibration) with flight telemetry (position, altitude, and wind) into a unified dashboard would enable continuous environmental monitoring and dynamic routing. This parallels connected perfusion platforms such as XVIVO Insights, which allow remote observation of perfusion parameters. Unified telemetry between UAVs and perfusion consoles could provide near‐real‐time oversight of both flight and graft status, improving synchronization between retrieval and implantation teams.

### 4.2 Infrastructure Requirements

Sustained UAV‐based organ delivery will depend on coordinated hospital, airspace, and digital infrastructure. At the hospital level, sites will need purpose‐built or adapted vertiports—infrastructure designed for vertical take‐off and landing—located close to theatres or transplant hubs to minimize hand‐off time. These should meet aviation standards for clearance and access control, with sterile, temperature‐controlled loading bays allowing direct transfer between perfusion consoles and UAV payloads.

Each site will require dedicated charging stations with battery‐swap capability, integrated weather sensors, and automated flight‐authorization systems to support continuous operation. A designated logistics coordinator should oversee scheduling, payload preparation, and telemetry validation. Reliable connectivity between theatre schedules, transplant registries, and dispatch platforms will be essential to avoid idle time and ensure readiness for rapid launch.

Vertiports and data hubs should be designed with redundancy and resilience in mind. Uninterruptible power supply (UPS) units, backup communications, and manual‐override capability are essential for continuity of service. Hospital pads must incorporate fire‐suppression systems, designated emergency‐landing areas, and acoustic shielding to comply with safety and environmental standards.

Experience from Rwanda's blood‐delivery network confirms that sustained operation depends as much on local training, maintenance, and spare‐part availability as on aircraft performance itself. Emerging systems such as Switzerland's Jedsy platform, which enables drones to dock directly onto hospital windows for automated exchange and charging, illustrate how infrastructure can be integrated seamlessly within existing healthcare facilities and urban environments [40].

### 4.3 Digital Integration and Operational Workflow

Digital architecture will underpin system reliability. Each mission should generate a verifiable telemetry stream linking allocation data, environmental parameters, and flight performance. Integration between UAV telemetry and perfusion systems—such as XVIVO Insights—would allow clinicians to track graft condition in real time throughout transit.

A cloud‐based dispatch network could match available drones to organ offers, assign optimal routes, and provide live arrival estimates to surgical teams. Interoperable application‐programming interfaces (APIs) linking UAV software, transplant registries, and electronic medical records would enable predictive scheduling and reduce manual handovers. Reliable encryption and authentication are critical to maintain clinical‐grade data integrity and auditability.

In practical terms, a mature UAV transport ecosystem could operate as an “Uber for organs**”**—an on‐demand, digitally orchestrated logistics grid akin to automated dispatch systems in emergency medicine. Such a system would autonomously deploy the nearest certified aircraft as soon as a retrieval is confirmed. Theatre teams could receive live notifications of location, temperature stability, and estimated arrival, allowing parallel preparation and minimizing cold‐ischemia exposure.

### 4.4 Reliability, Maintenance, and Workforce

Reliability depends not only on airframe performance but also on robust ground infrastructure and trained personnel. Routine pre‐flight inspection, battery‐health monitoring, and post‐mission diagnostics should be standardized across networks. Automated fault‐detection and sensor‐calibration systems can reduce downtime and ensure compliance with aviation safety standards.

Workforce readiness is equally critical. Logistics coordinators, retrieval teams, and transplant staff must receive hands‐on training in UAV operation, infection‐control procedures at loading sites, and emergency‐abort protocols. Simulation‐based drills linking aviation and surgical teams could strengthen interdisciplinary coordination.

Long‐term maintenance contracts and centralized parts supply will be vital for reliability. Drawing from established air‐ambulance and Zipline‐style medical networks, sustained uptime will rely on local technical capacity, frequent inspection, and clearly defined service intervals.

### 4.5 Operational and Business Model Frameworks

Translating proof‐of‐concept flights into a sustainable clinical service will require viable operational and funding models. Most early programs have adopted public–private partnerships (PPPs) linking hospitals, aviation operators, and innovation agencies to share infrastructure costs and ensure compliance. In the United States, for instance, the University of Maryland group established *MissionGO* and *MediGO* to collaborate with Organ Procurement Organizations such as LifeSource and the Nevada Donor Network, while in the United Kingdom, *Apian*, *Skyports*, and *CAELUS* have secured UKRI funding to develop NHS‐integrated drone corridors. These frameworks illustrate how publicly funded infrastructure combined with private‐sector technical management can accelerate safe deployment.

Future scalability may follow one of two models:

1. Centrally managed regional fleets, akin to national air‐ambulance networks, ensuring equitable coverage and consistent safety oversight; or
2. Accredited vendor networks, where certified UAV operators deliver on‐demand logistics as subscription or per‐mission services.

Both models will require defined reimbursement pathways and liability structures for payloads, operators, and healthcare providers. Ultimately, whichever approach prevails, early clarity on governance, liability, and reimbursement will determine whether UAV logistics can transition from demonstration to dependable clinical utility.

A fully developed UAV‐enabled transplant network would operate as a digitally orchestrated, high‐reliability system—autonomous in flight, closely monitored on the ground, and seamlessly integrated into clinical workflows with precise environmental control and robust redundancy. Through standardized vertiport design, integrated telemetry, and continuous staff training, these technologies could transform organ transport from a fragile logistical bottleneck into a predictable, auditable, and life‐saving extension of the modern transplant pathway.

## 5 Regulatory, Legal, and Ethical Readiness

The transition from feasibility to clinical implementation of drone‐based organ delivery is shaped less by technical constraints than by the absence of a coherent legal and regulatory framework. Proof‐of‐concept flights have demonstrated technical reliability and traceability, yet governance mechanisms have not evolved in parallel. Under current regulations, aviation liability rests with drone operators and their insurers, while Organ Procurement Organizations (OPOs) and transplant centers remain responsible for organ custody. The introduction of autonomous or semi‐autonomous systems disrupts these traditional divisions of responsibility, raising unresolved questions around accountability, insurance, and indemnity. Should transplant centers bear shared operational risk for unmanned flights despite lacking control over them? How should liability be allocated among corridor operators, software vendors, and healthcare providers in the event of telemetry or communication failure? The absence of an integrated governance framework for unmanned medical logistics continues to impede large‐scale adoption

### 5.1 Airspace Regulation and Classification

In both the United States and the United Kingdom, strict regulatory limits currently constrain *beyond‐visual‐line‐of‐sight* (BVLOS) medical drone operations—flights that extend beyond the pilot's direct line of sight and therefore require advanced tracking and safety systems.

In the United States, uncrewed aerial vehicle (UAV) flight is regulated under 14 CFR Part 107, which limits altitude to below 400 ft, requires continuous visual oversight, and prohibits the carriage of hazardous materials [41]. To exceed these limits, operators must obtain a *Part 107 Waiver* supported by detailed safety documentation and risk assessments [41]. The 2019 University of Maryland kidney flight, for example, operated under such waivers with police coordination and temporary road closures to ensure safety [20]. Broader permissions for compensated BVLOS medical deliveries fall under Part 135 Air Carrier Certification, which imposes strict safety‐management and pilot‐training standards [42]. UAVs are not yet classified as emergency aircraft and therefore cannot request priority access to controlled airspace [43].

In the United Kingdom, UAV operations fall under Civil Aviation Authority (CAA) CAP 722, where organ transport is classed as a “Specific Category” activity. Operators must submit an operational safety case—often derived from the Specific Operations Risk Assessment (SORA)—and obtain formal authorization for beyond‐visual‐line‐of‐sight (BVLOS) flight. Aircraft are required to meet airworthiness and maintenance standards comparable to those in commercial aviation, incorporating reliable detect‐and‐avoid sensors and encrypted control links. Automated safety features must behave predictably—returning to base or landing safely in the event of communication loss—so that autonomous decision‐making cannot endanger people or property [44]. A recent European trial by Quintanilla et al. [45] demonstrated successful urban medical drone flights under this SORA‐based framework, showing that while compliance pathways exist, harmonized policies and healthcare‐specific guidance are still required to enable routine operations within controlled airspace.

### 5.2 Legal Accountability and Governance

Organ transport already spans overlapping jurisdictions among OPOs, transplant centers, and regulators. UAV operations add a new operational layer. Under current law, primary liability lies with the operator and insurer, while hospitals and OPOs retain secondary responsibility for graft integrity. The semi‐autonomous nature of UAVs complicates conventional accountability, particularly after technical or communication failures.

In the United Kingdom, the Human Tissue Act 2004 (HTA) defines organ retrieval, storage, and transport as licensable activities overseen by the Human Tissue Authority. Any organization moving organs—including drone operators—must hold or operate under an HTA license [46]. Section 16(7) exempts “storage incidental to transportation,” but full traceability and validated temperature monitoring remain mandatory. Comparable oversight exists in the United States, where the Food and Drug Administration (FDA) regulates human tissue handling under *21 CFR §1271*, and the Health Resources and Services Administration (HRSA) oversees OPOs via the United Network for Organ Sharing (UNOS). Accountability for transport safety is largely contractual rather than statutory, and liability definitions remain inconsistent.

If a UAV failed mid‐flight, responsibility among the operator, manufacturer, OPO, and transplant center would be ambiguous. Insurers are now developing hybrid aviation‐medical policies that include payload coverage, though definitions of negligence and exclusion thresholds vary [26]. Parallels exist with telemedicine, where distributed liability is managed through contractual delineation and shared‐governance agreements. In the near term, governance will depend on contractual clarity—defining liability, operational control, and incident‐reporting obligations.

Future frameworks include shared‐liability models, allocating risk proportionally among UAV providers, transplant centers, and coordination bodies. Contracts should align with HTA and FDA/HRSA quality systems and include procedures for Serious Adverse Event and Reaction (SAEAR) reporting. As autonomy increases, questions of “reasonable foreseeability” and duty of care will require formal legal interpretation. Ethical considerations will also include whether recipients should be informed that their organ was transported by drone, and how telemetry or location data are recorded in transplant documentation.

Ultimately, UAV governance must balance innovation with patient safety and transparency, preserving the standards of traceability, documentation, and professional accountability that underpin all transplantation.

### 5.3 Cybersecurity and Data Protection

As drones integrate into clinical logistics, the security of navigation and data systems becomes critical. Each flight depends on satellite positioning, radio communication, and cloud‐based telemetry—each a potential target for interference. Although no breach has yet affected organ missions, studies in the broader UAV field confirm vulnerability. Khan et al. [47] demonstrated that civilian GPS signals can be manipulated with inexpensive tools; Ferrão et al. [48] replicated this experimentally and proposed “sanity‐check” algorithms to trigger automatic failsafes. For medical missions, such spoofing could misroute organs or corrupt tracking, invalidating the clinical chain of custody.

These risks also fall under the healthcare data‐protection law. In the United States, the Health Insurance Portability and Accountability Act (HIPAA) mandates that any patient‐identifiable data sent or stored electronically be encrypted using recognized standards such as AES‐256 and Transport Layer Security (TLS 1.3) [49]. Properly encrypted data are not considered reportable breaches under HIPAA. In Europe, the General Data Protection Regulation (GDPR) imposes equivalent obligations: any data linked to a donor or recipient must be securely transmitted and stored, with strict access controls and audit trails to ensure confidentiality and traceability.

In practice, communications between aircraft, ground control, and cloud servers must be encrypted end‐to‐end with verifiable audit trails. Devices such as Scalea's Human Organ Monitoring and Quality Apparatus for Long‐Distance Travel (HOMAL) system—which record temperature, vibration, and location—handle indirectly identifiable data and must therefore be encrypted, access‐controlled, and retained only as long as clinically necessary.

Security extends beyond data. The review by Stierlin et al. [50] highlights that redundant communication (including 5G), AI‐based navigation, and tamper‐evident containers now underpin UAV safety. Smart payloads capable of temperature and humidity regulation, coupled with blockchain‐based traceability, enhance both sample integrity and legal accountability. Wildlife‐interaction risk remains low for the small altitudes and durations typical of medical flights, but designated corridors and radar‐based detect‐and‐avoid systems are recommended to mitigate bird‐strike hazards.

Cybersecurity in UAV organ transport is inseparable from clinical safety. A data breach or navigation failure could delay delivery, corrupt traceability, or expose confidential information. Secure encryption, authentication, redundant systems, and compliance with HIPAA and GDPR are therefore essential clinical obligations, not optional safeguards.

### 5.4 Ethical and Social Dimensions

Ethical issues extend beyond regulation to consent, transparency, and equity. Patients are seldom informed of the precise transport method used for their graft, yet the introduction of autonomous systems raises new expectations. In the Toronto lung trial, Sage et al. [21] obtained explicit consent acknowledging the novelty of UAV transport, but in future, general consent at listing may suffice once regulated use is established. Public confidence will depend on transparent safety reporting and a phased rollout rather than isolated pilot demonstrations.

Equity remains crucial. Drones can shorten transport routes in remote or infrastructure‐poor regions, improving access for rural populations [19]. In sub‐Saharan Africa and South Asia, UAVs already deliver blood and diagnostics within minutes, demonstrating potential to reduce geographic disparities [6]. Without deliberate policy, however, early adoption could favor urban centers.

Public perception also matters. Drones retain military associations, and concerns about safety, noise, and privacy persist. These can be mitigated by community engagement, visual identification of medical aircraft, and clear separation from defense use [19]. Transparent reporting, visible oversight, and consistent safety performance will build trust.

Professional safety is another ethical dimension. Organ‐retrieval travel remains one of the riskiest duties in transplantation—six members of a University of Michigan procurement team were killed in a 2007 plane crash en route to a retrieval. Replacing such missions with uncrewed systems could significantly reduce occupational risk [20].

In summary, ethical readiness for drone‐based organ transport rests on three principles: transparency, ensuring professionals and patients understand the technology's purpose and limits; equity, extending benefits to rural and resource‐limited areas; and trust, built through consistent safety, community engagement, and protection of both donor privacy and clinician welfare. Addressing these considerations early will be essential to align UAV innovation with the humanitarian ethos of transplantation.

## 6 Economic and Environmental Considerations

Conventional organ transport methods vary widely in cost, reliability, and environmental footprint. Table 3 summarizes the main logistical characteristics and trade‐offs between drones and existing transport modes.

**TABLE 3:** Comparison of drone transport with conventional methods.

Transport mode	Typical range	Main sources of delay	Predictability	Relative cost	Environmental impact
Road (ambulance or courier van)	Short–medium	Traffic congestion, driver availability, after‐hours delays	Variable	Low‐medium	Moderate‐high (fuel use, congestion)
Commercial airline	Long	Flight scheduling, baggage handling, security checks, weather	Low	Low (per km)	High (jet fuel emissions)
Helicopter or charter aircraft	Medium‐long	Crew duty limits, weather, air‐traffic control	Medium	Very high	High
Drone (uncrewed aerial vehicle)	Short–medium (≤20 km current; >100 km under development)	Weather, battery endurance, airspace restrictions	High (direct point‐to‐point routing)	Low‐medium	Low (electric propulsion, minimal emissions)

*Note:* Comparison of key logistical parameters—speed, range, cost, environmental impact, and operational reliability—across traditional organ‐transport methods (road, helicopter, fixed‐wing aircraft) versus emerging UAV systems. Values represent typical reported or modeled ranges rather than fixed benchmarks.

### 6.1 Economic Feasibility Within Health‐System Logistics

Although improved clinical outcomes provide the primary rationale for UAV‐enabled organ transport, long‐term adoption will depend on whether such systems are financially sustainable and operationally efficient within publicly funded health services. Economic evaluation, therefore, provides context for scalability rather than justification in itself.

Conventional organ transport is both costly and operationally inconsistent. Ground transfers, though inexpensive (≈ USD 118 per organ) [4], require dedicated drivers and vehicles and remain vulnerable to congestion, staffing shortages, and after‐hours delays. Air transport reduces these uncertainties but at a markedly higher cost. Typical cost estimates vary by region and provider: helicopter transfers are commonly reported around USD 10–15 k per mission, fixed‐wing aircraft between USD 7–10 k, and private charters up to USD 40 k or higher [20].

Early demonstrations suggest that UAVs could deliver comparable reliability at a fraction of these costs. Mid‐America Transplant reported that transporting donor blood samples by aircraft cost ≈ USD 10 000, whereas equivalent drone delivery cost ≈ USD 1000 [51]. Likewise, the Matador UAS Consortium—a partnership between Donor Alliance and Texas Tech University—found that drone deliveries operated at roughly one‐tenth the cost of conventional methods (Donor [52]). These figures, however, exclude fixed costs such as aircraft procurement, certification, infrastructure, maintenance, and training.

A realistic appraisal, therefore, requires whole‐system modeling that includes both capital and recurrent expenditure—aircraft acquisition, maintenance, insurance, charging infrastructure, and workforce training—offset against expected flight volumes. Integration into existing allocation systems such as the United Network for Organ Sharing (UNOS) or NHS Blood and Transplant (NHSBT) could allow UAV logistics to be reimbursed within standard organ‐acquisition fees, supporting cost‐sharing rather than parallel contracting. Formal budget‐impact analyses should report costs both per mission and per organ transplanted.

### 6.2 Environmental Performance and Long‐Term Sustainability

While financial sustainability is essential, environmental performance is an equally important determinant of long‐term viability. Conventional aviation remains one of the most carbon‐intensive components of transplant logistics, with single retrieval flights emitting several hundred kilograms of CO_2_. Electric UAVs, in contrast, consume only a few kilowatt‐hours per flight and produce negligible direct emissions when charged using low‐carbon electricity.

Empirical data from NHS medical‐drone pilots illustrate these gains. Replacing four short‐range van routes with UAVs in southern England reduced CO_2_ emissions by ≈ 60% while maintaining delivery reliability [53, 54]. Broader modeling indicates that operational emissions from small electric UAVs are typically an order of magnitude lower than those of ground vehicles performing equivalent medical deliveries [55, 56]. These savings arise primarily from avoided vehicle mileage and low per‐flight energy demand.

Lifecycle analyses, however, temper these advantages. The manufacture, charging, and disposal of lithium‐ion batteries contribute significantly to embodied emissions. Rodrigues et al. [57] and Kumar et al. [58] estimate that production and replacement of drone batteries may offset 10%–30% of operational savings, depending on flight frequency and duty cycle. Battery degradation and recycling efficiency therefore critically influence overall sustainability. Propeller noise, although far lower than helicopter levels, also requires consideration for community tolerance and may necessitate operational limits in dense urban areas.

The most credible sustainability contribution of UAVs lies in improving system efficiency rather than achieving absolute carbon neutrality. By preventing redundant journeys, cancellations, and sub‐optimal routing, autonomous corridors can reduce wasted resources while maintaining graft viability. When paired with renewable‐powered charging hubs, drone networks could deliver incremental yet measurable environmental gains without major new infrastructure. Within transplantation logistics, these cumulative efficiencies may prove as valuable as direct emission reductions in building a resilient, low‐carbon transport ecosystem.

## 7 Public and Professional Readiness

### 7.1 Professional Confidence and Clinical Readiness

The routine adoption of drone‐enabled organ transport will depend on whether clinicians, coordinators, patients, donor families, regulators, and the public trust the technology—and whether healthcare systems are equipped to support it. While most stakeholders recognize the potential for logistical improvement, they also demand firm assurances of safety, reliability, liability, and workflow compatibility.

Among transplant professionals, support remains conditional. In a survey of US transplant surgeons, 92.7% believed drones could improve organ transport, yet most rated their knowledge as “average or below average.” Approximately 23% reported feeling fearful about drones, and 34.5% said civilian drones made them nervous [59]. This mixture of optimism and anxiety reflects both enthusiasm for innovation and caution, shaped by past transport failures. Structured training, simulation, and transparent incident reporting will be essential to convert theoretical confidence into practical readiness. Early adopters in Baltimore and Toronto reported that initial skepticism shifted to advocacy once successful kidney and lung deliveries were observed in practice [20, 21].

To embed drones within existing clinical logistics, hospitals must ensure that staff understand chain‐of‐custody procedures, packaging standards, and emergency protocols. Integration with electronic medical records (EMRs) and allocation platforms such as UNOS or NHS Blood and Transplant (NHSBT) will require clear workflows, secure data exchange, and delineated accountability for launch, monitoring, and receipt. Practical measures—RFID tagging, tamper‐evident seals, and synchronized temperature or perfusion logging linked to UAV telemetry—will allow clinicians to audit each delivery without assuming new legal risk. Professional indemnity frameworks should explicitly clarify that responsibility for flight operations remains with certified operators rather than surgical or retrieval teams.

### 7.2 Public Perspective

At a societal level, attitudes remain mixed but conditionally supportive. A US Postal Service survey found 44% viewed drones favorably, 34% unfavorably, and 23% were undecided; only 32% considered them safe, with malfunction risk the chief concern [60]. Cross‐national studies show similar patterns: in Germany, 30%–40% supported drone delivery, but 91% cited criminal misuse, 86% privacy, and 53% noise as concerns; acceptance was higher in rural (68%) than urban (30%) settings (Eißfeldt et al. [61]). The “Not Over My Own Home” (NOMOH) effect remains evident—hovering drones increased annoyance scores from 2.3 to 6.8 on an 11‐point scale in quiet environments (Torija et al. [62]). Acceptance thus depends less on abstract attitudes than on local context, perceived purpose, and acoustic exposure. Corridors aligned with existing traffic noise and limited to daytime operations may reduce resistance.

Public confidence hinges on transparency and framing. Portraying UAVs as clinical tools that extend healthcare reach elicits greater support than commercial or surveillance narratives [63]. Hospitals introducing drone logistics should provide clear communication on routes, altitudes, hours, and safety reporting channels. Early engagement with patients, donor organizations, and local communities will be key to building lasting trust.

## 8 Conclusion and Future Directions

Drone‐assisted organ transport has progressed from concept to credible adjunct within modern transplant logistics. Early demonstrations across multiple continents confirm that uncrewed aircraft can deliver organs safely, maintaining stable temperature and vibration profiles without graft injury. The clinical rationale remains clear—reducing avoidable delay can preserve viability and expand utilization—but future work must now move beyond proof of principle toward measurable system value.

The current evidence base remains preliminary. Most flights have been short‐range (<5 km) and manually operated under visual line‐of‐sight, constrained by payload capacity, battery life, and regulation. The next phase must focus on comparative trials embedded in real transplant workflows, directly measuring effects on cold‐ischemia time, delayed graft function, discard rate, and cost. Smaller payloads such as blood, cross‐match, or biopsy samples provide safe early domains for validating telemetry accuracy, cybersecurity, and traceability before full organ deployment.

Regulatory evolution will be decisive. Experimental waivers must give way to certified medical UAV corridors with predictable beyond‐visual‐line‐of‐sight (BVLOS) permissions, harmonized insurance standards, and transparent reporting. National agencies—FAA, CAA, NHSBT, HTA—should align requirements for airworthiness, data security, consent, and incident response.

Interoperability is equally critical. Hospital systems will require standardized pads, charging hubs, and contamination‐controlled hand‐off areas, supported by unified telemetry and EMR integration so that UAV flight data, allocation records, and perfusion parameters form a single auditable dataset.

Workforce and governance readiness must advance in parallel. Coordinators, perfusionists, and OPO staff need simulation‐based training, clear indemnity frameworks, and defined escalation pathways for technical or legal failures. Ethical guidance should clarify consent language, data stewardship, and patient communication.

Finally, adoption should be equity‐led—targeting regions where existing logistics most often fail—while contributing to a more resilient, low‐carbon transport network.

Table 4 summarizes the key research and policy questions that must be addressed to translate drone delivery from feasibility to routine clinical integration.

**TABLE 4:** Priority areas for future research on drone‐assisted organ transport.

Domain	Key research questions
1. Time sensitivity and clinical evidence	How do drone deliveries compare with road or air transport in terms of total travel time, cold ischemia duration, and transplant outcomes? Which organ types and journey distances show the greatest clinical benefit from drone use?
2. Integration with organ perfusion and preservation technologies	Can drones safely transport perfused organs while maintaining stable temperature, vibration, and power supply? Could linking perfusion monitoring systems with drone telemetry improve coordination and surgical readiness?
3. Technological, infrastructural, and operational readiness	What range, payload capacity, and landing infrastructure are required for reliable hospital‐to‐hospital delivery? How can flight data, hospital tracking, and safety systems be integrated to ensure full traceability and cybersecurity?
4. Regulatory, legal, and ethical readiness	What airspace and safety regulations are needed to permit routine long‐distance medical drone operations? Who holds legal responsibility in the event of transport failure—the healthcare provider, operator, or manufacturer?
5. Economic and environmental considerations	Are drones cost‐effective compared with ambulances or helicopters when factoring in equipment, staffing, and maintenance costs? What is the overall environmental impact of drone transport, including electricity use, battery production, and recycling?
6. Public and professional readiness & system integration	How can training and standard operating procedures build professional confidence in drone use for organ transport? How can healthcare organizations communicate with patients, donors, and the public to build trust and acceptance of drone logistics?

*Note:* Summary of major research and policy questions identified through thematic synthesis of the reviewed literature. These priorities span six domains—clinical evidence, integration with perfusion technology, operational readiness, regulatory and ethical frameworks, economic and environmental assessment, and public or professional preparedness—intended to guide future multidisciplinary investigation and system design.

UAVs should be viewed not as replacements for current systems but as integrative technologies enhancing the reach, reliability, and sustainability of transplantation. Their success will be measured not by novelty, but by the organs preserved, lives extended, and trust maintained across the global transplant community.

## Author Contributions

Robson G. Gilmour designed the study and conducted the literature review, planned, structured, drafted, and wrote the manuscript. Mekhola Hoff provided the initial idea for the literature review, acted as a mentor and guide for the study, reviewed multiple drafts of the manuscript, and provided feedback and opinions on key discussions.

## Funding

The authors have nothing to report.

## Conflicts of Interest

The authors declare no conflicts of interest.

## Data Availability

Data sharing is not applicable to this article as no datasets were generated or analyzed during the current study.
